# Dietary intervention among young cancer survivors within the CARE for CAYA program

**DOI:** 10.1007/s11764-026-01997-4

**Published:** 2026-03-26

**Authors:** Julia von Grundherr, Simon Elmers, Luisa Samland, Raika Mühlberg, Sarah Dwinger, Jannike Baumann, Barbara Koch, Franziska Wolters, Lesley-Ann Hail, Gabriele Escherich, Corinna Bergelt, Wiebke Jensen, Eik Vettorazzi, Hans Pinnschmidt, Luzia Valentini, Freerk T. Baumann, Susanne Singer, Rüdiger Reer, Claudia Metz, Gabriele Calaminus, Jörg Faber, Dana Schmidt, Thorsten Langer, Inken Hilgendorf, Michael Koehler, Alexander Puzik, Madeleine Aschhoff, Annette Sander, Lisa Schiffmann, Magdalena Sokalska-Duhme, Sonja Schuster, Ann-Kristin Kock-Schoppenhauer, Martin Smollich, Matthias Rostock, Carsten Bokemeyer, Alexander Stein, Marianne Sinn

**Affiliations:** 1 Department of Oncology, Hematology, BMT with Section Pneumology, Hubertus Wald Tumor Center, University Cancer Center Hamburg, University Medical Center Hamburg-Eppendorf, Martinistr. 52, 20246 Hamburg, Germany; 2 Department of Pediatric Hematology and Oncology, Hubertus Wald Tumor Center, University Cancer Center Hamburg, University Medical Center Hamburg-Eppendorf, Hamburg, Martinistr. 52, 20246 Germany; 3 Department for Medical Psychology, University Medical Center Hamburg-Eppendorf, Martinistr. 52, Hamburg, 20246 Germany; 4 Institute for Medical Psychology, Greifswald University Medicine, Walther-Rathenau-Straße 48, Greifswald, 17475 Germany; 5 Institute of Medical Biometry and Epidemiology, University Medical Center Hamburg-Eppendorf, Martinistr. 52, Hamburg, 20246 Germany; 6 University of Applied Sciences, Faculty of Agriculture and Food Sciences, Brodaer Straße 2, Neubrandenburg, 17033 Germany; 7 Department I of Internal Medicine, Center for Integrated Oncology Aachen Bonn Cologne Dusseldorf, University Hospital of Cologne, Kerpener Str. 62, Cologne, 50937 Germany; 8 Department Quality of Life, Comprehensive Cancer Center—Mecklenburg-Vorpommern, University Medical Center Rostock, Rostock, 18057 Germany; 9 Institute of Human Movement Science, Faculty of Psychology and Human Movement, University of Hamburg, Turmweg 2, Hamburg, 20148 Germany; 10 Clinic for Pediatrics III, Department of Pediatric Hematology/Oncology, West German Cancer Centre, University Hospital Essen, Essen, Hufelandstraße 55, Germany; 11 Pediatric Hematology and Oncology, University Hospital Bonn, Venusberg Campus 1, Bonn, Germany; 12 Department of Pediatric Hematology/Oncology, Center for Pediatric and Adolescent Medicine – University Cancer Center (UCT), University Medical Center of the Johannes Gutenberg-University Mainz, Langenbeckstraße 1, Mainz, 55131 Germany; 13 Universitätsmedizin Rostock, Univ.-Kinder- und Jugendklinik Rostock, Ernst-Heydemann-Str. 8, Rostock, 18057 Germany; 14 Pediatric Oncology, Children’s Hospital, University Hospital of Schleswig-Holstein, Campus Luebeck, Ratzeburger Allee 160, Luebeck, 23538 Germany; 15 Klinik für Innere Medizin II, Abteilung für Hämatologie und Internistische Onkologie, Universitätsklinikum Jena, Am Klinikum 1, Jena, 07747 Deutschland; 16 Department of Hematology and Oncology, University Hospital Magdeburg, Leipziger Str. 44, Magdeburg, 39120 Germany; 17 Specialty Practice for Psycho-Oncology, Magdeburg, Hegelstr. 4, Magdeburg, 39104 Germany; 18 Department of Pediatric Hematology, Oncology and Stem Cell Transplantation, Children’s Hospital, Medical Center – University of Freiburg, Faculty of Medicine, University of Freiburg, Breisacher Str. 62, Freiburg, D-79106 Germany; 19 University Hospital Muenster, Medical Clinic B for Gastroenterology, Hepatology, Endocrinology, Clinical Infectiology, Department for Endocrinology, Diabetology and Nutritional Medicine, Albert-Schweitzer Campus 1, Muenster, 48149 Germany; 20 Medizinische Hochschule Hannover, Klinik für Pädiatrische Hämatologie-Onkologie, Carl-Neuberg-Str. 1, 30625 Hannover, Germany; 21 Institute of Clinical Epidemiology and Biometry, Julius Maximilians-Universität Würzburg, Würzburg, Germany; 22 CCC WERA, University Hospital Würzburg, Josef-Schneider-Str. 6, Würzburg, 97080 Germany; 23 Department of Pediatric Hematology, Oncology & Immunology, Olgahospital, Klinikum Stuttgart, Kriegsbergstrasse 62, Stuttgart, 70174 Germany; 24 Abteilung für pädiatrische Hämatologie und Onkologie, Kinder- und Jugendklinik, Universitätsklinikum Erlangen, Loschgestr. 15, Erlangen, 91054 Germany; 25 Institute of Medical Biometry and Statistics, Section for Clinical Research IT, University of Luebeck and University Hospital Schleswig-Holstein, Ratzeburger Allee 160, Luebeck, 23562 Germany; 26 Institute of Nutritional Medicine, University Hospital Schleswig-Holstein, Luebeck, 23538 Germany

**Keywords:** CAYA cancer survivor, Dietary quality, Quality of life, Fatigue, Nutritional counseling, Mediterranean dietary adherence

## Abstract

**Purpose:**

A healthy diet and weight can reduce the risk of secondary diseases in child, adolescent, and young adult (CAYA) cancer survivors.

**Methods:**

The *nutrition module* of the CARE for CAYA program, a multimodal survivorship program, implemented needs-based dietary interventions for CAYAs (aged 15–39 years) at 14 German university clinics between December 2017 and July 2020 (RCT: January 2018 to July 2019). Individual needs were determined at baseline (T1), and those with *high need for dietary intervention* received intensified intervention (intervention group (IG)), while the control group (CG) received one basic counseling. Dietary quality was measured after 52 weeks (T3) using the HEI-EPIC. Changes in Mediterranean dietary adherence (MEDAS-FFQ), phase angle, BMI, nutrition-related barriers, fatigue, and quality of life, as well as subjective and objective taste perception were assessed.

**Results:**

Overall, 74.4% (*n* = 267; 134 IG; 133 CG) had a high need for dietary intervention. Median age was 24.8 (interquartile range 19.6–31.6) and 66.7% were female. The dietary quality improved in IG (T1 = 50.0 pts; T3 = 58.0 pts) and in CG (T1 = 49.0 pts; T3 = 57.5 pts, intergroup difference *p* = 0.773). The Mediterranean dietary adherence improved significantly (IG) (*p* = 0.040). Both groups had improvements in fatigue and quality of life, empowerment for lifestyle changes, and high satisfaction with the intervention.

**Conclusion:**

The dietary intervention within a multimodal program had a positive impact on dietary quality and mental health in CAYAs.

**Implications for Cancer Survivors **

These findings may guide the development of future (dietary) programs in the follow-up care of CAYA cancer survivors.

**Trial registration**: The trial was registered with the German Clinical Trial Register on 19 January 2018 (DRKS00012504|http://www.germanctr.de/).

**Supplementary Information:**

The online version contains supplementary material available at https://doi.org/10.1007/s11764-026-01997-4.

## Introduction

Each year, approximately 500,000 patients are diagnosed with cancer in Germany, of whom about 4% are aged between 0 and 39 years [1], defined as CAYAs (Children, Adolescents and Young Adults) [2]. Multimodal therapies have improved the survival rate for CAYAs to over 80% [3]. However, CAYAs are at increased risk of long-term sequelae, including physical problems [4], cardiovascular events [5], as well as psychosocial challenges [4, 6], and a reduced quality of life (QoL) [7, 8].

To address challenges of young cancer survivors, the *CARE for CAYA Program (CFC-P)*, including a multicenter randomized controlled trial (RCT), was developed as Germany’s first multimodal follow-up care program to determine the efficacy of respective need-based interventions in nutrition, physical activity, and psychosocial needs [9]. The results showed that up to 85% had a high need for intervention(s). Although the interventions could not reduce the overall rate of high need in participants after 52 weeks [10], both groups reported reduced self-reported needs, improved QoL, less fatigue, and a high satisfaction rate with the CFC-P.

The findings from the CFC-P underscore the complexity of CAYAs’ needs and highlight the need for further intervention research, but also with regard to the different modules. Although a healthy lifestyle may reduce the risk for cardiovascular events [11] and improved health-related QoL [12], studies already revealed that a considerable proportion of CAYAs do not engage in a balanced diet [13–15]. Up to 65% of cancer survivors faced nutrition-related side effects, 74% received no dietary intervention, and 32% reported worsened dietary quality since diagnosis [16].

The most commonly used instruments in nutritional research are food diaries, 24-h recalls, and Food Frequency Questionnaires (FFQs), as well as dietary quality indices such as the Healthy Eating Index (HEI) (USA) and the HEI-EPIC (European Prospective Investigation into Cancer and Nutrition) (Germany). At the time of the CARE for CAYA program, one validated instrument was available in Germany: the HEI-EPIC questionnaire [17], developed in the early 2000s as part of the EPIC study [18]. None of these instruments was suited for young cancer survivors, and few provided a comprehensive picture of dietary quality or reflected the current state of research [19–25]. These approaches are methodologically demanding and time-consuming, involving extensive data processing and complex nutrient or food group calculations [26].

Overall, identifying and promoting healthy dietary patterns is crucial to improve long-term health of cancer survivors [27]. While personalized nutritional counseling may improve dietary behavior in CAYAs [13], evidence on its effectiveness remains limited. This manuscript presents data from the *nutrition module* of the CFC-P.

## Materials and methods

### Study design of the overall CFC-P and the CFC-nutrition module

The CFC-P was an interventional multicenter randomized two-arm parallel group clinical trial (RCT; January 2018 to July 2019) (see CONSORT reporting guidelines Table A1 [28]) for young cancer survivors to address needs in the modules* nutrition, physical activity, and psycho-oncology.* Participants with high need in at least 1 module were randomized to intervention group (IG) and control group (CG). The study included a 1:1 allocation and was embedded within an ongoing longitudinal cohort study (July 2019 to March 2021) at 14 university outpatient clinics in Germany, funded by the Innovation Fund of the Federal Joint Committee (GBA) [9, 10]. At the consortium leadership’s request, randomization was performed using a concealed, block-randomized, computer-generated list, stratified by site. The results were communicated to the sites via telephone and fax. Due to the nature of the intervention, it was not possible to blind the participants or the study personnel. Although no adverse events or harms were expected during the study period, any such events would have been systematically recorded due to the design of the study. CAYAs with former malignancies (ICD-10 codes C00.Z to C97.Z) could be included.

After a comprehensive assessment, individual high needs in the fields of nutrition, physical activity, and psycho-oncology at baseline (T1) and after 52 weeks (T3) were determined, and participants could take part in up to three modules. Participants with no high need were offered one basic counseling session in each module. The defined *high need criteria* for the CFC-nutrition module were: (a) patient self-reported outcomes (PRO) of a poor dietary quality (≤ 40 Healthy Eating Index—European Prospective Investigation into Cancer and Nutrition (HEI-EPIC) score) [29] or (b) medical examinations as underweight (< 18.5 body mass index (BMI)) [30], gastrointestinal symptoms, or criteria for metabolic syndrome [9, 10].

The intervention group (IG) of the CFC-nutrition module received assessments and nutritional counseling based on the standardized German-Nutrition Care Process (G-NCP) [31]. This included a nutritional assessment, diagnosis, intervention, monitoring, and evaluation [31].

The CFC-nutrition intervention aimed to provide a personalized counseling and helped CAYAs identify and overcome their individual nutrition-related barriers. It consisted of five personalized appointments (30–45 min) every 6 weeks for a period of 24 weeks. The dietary-focused questionnaires (see Sect. “Study outcomes”) and an assessment questionnaire were used to evaluate the dietary quality and related factors, such as restrictions or problems. Based on this assessment, individual nutritional recommendations were made. In the follow-up appointments, the implementation of individual recommendations and current nutrition topics were discussed. Additional modular assessments (e.g., bioelectrical impedance analysis (BIA)) were implemented to improve participant compliance. CAYAs received general and personalized newsletters. They were invited to participate in a grocery shopping training (up to five participants; 45–60 min) and a cooking class (up to eight participants; 2–3 h) to further promote healthy eating. In contrast, the control group (CG) received a one-time counseling [9, 10]. Assessments and counseling sessions were conducted by nutritionists and dietitians trained by the consortium center, following a standardized procedure (pre-defined manual), and participated in quarterly calls to ensure ongoing exchange (Tidier-Checklist Table A2 [32]).

### Study outcomes

The primary outcome of the CFC-nutrition module was the improvement in the dietary quality (HEI-EPIC) [29] at 52 weeks (T3).

Secondary outcomes were phase angle (BIA), Mediterranean dietary adherence (Mediterranean Dietary Behavior Questionnaire (MEDAS-FFQ)) [33], nutrition-related barriers (Transtheoretical Model (TTM)—Step Algorithm for Nutrition (modified); Decision Balance Short Form (modified)) [34], BMI (World Health Organization (WHO) classification) [30], European Organization for Research and Treatment of Cancer Quality of Life Questionnaire (EORTC QLQ-C30) Global Health Score (GHS)/QoL and Fatigue Score [35], subjective (questionnaire by T. Hummel) [36], and objective taste perception (“taste strips”) [37]. In addition, satisfaction within the module was analyzed (ZUF-8) [38]. For more details regarding the study outcomes, see Table A3.

### Statistical analysis and statistical methods

For sample size calculation and statistical methods, see [9] and [10]. Analyses of the CFC-nutrition module were secondary endpoints of the CFC-P RCT with multiple timepoints and were exploratory in nature. Therefore, *p*-values should be interpreted descriptively. Descriptive statistics and categorical data were summarized by absolute and relative frequencies. Continuous data and intra-group comparison were reported as medians (quartiles). Effects were reported as absolute and relative changes with 95% confidence intervals. The two-sided significance level was set at 5%. Changes from T2 (week 16) to T1 and from T3 to T1 were analyzed as dependent variables, using baseline-adjusted general linear models with repeated measures. Fixed effects were time point, group, the interaction of time point and group, and the baseline value of the dependent variable at T1. Time points within patients were considered repeated measures. Model-estimated marginal means and their 95% confidence intervals (CI) were computed and compared across time points. Module analyses were performed intention-to-treat (ITT) by using SPSS version 29 (IBM Corp).

## Results

**Fig. 1:**
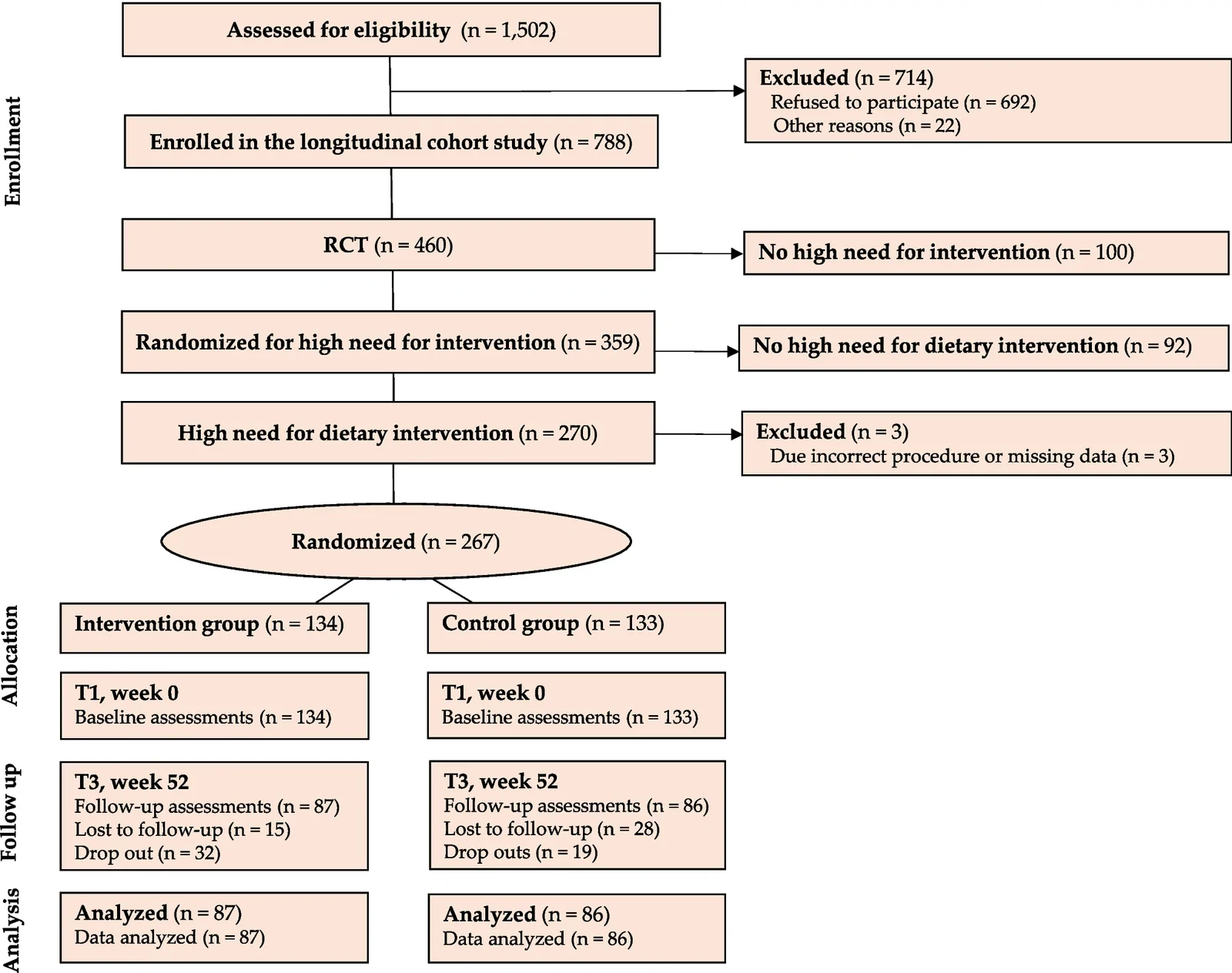
CONSORT participant flow diagram

In total, 1,502 CAYAs were screened for eligibility in the overall CFC-P, of whom 788 enrolled in the longitudinal cohort study. Of these, 460 CAYAs participated in the randomized controlled trial, involving 359 CAYAs with a *high need in at least one module* (not shown, please refer to [10]). Of the 359 randomized CAYAs, 270 had a high need for dietary intervention. Three CAYAs had to be excluded due to incorrect procedure or missing data. This corresponds to 267 CAYAs in the nutrition module of the CFC-P, representing 74.4% (267/359) of the randomized control trial: 134 (50.2%) in IG and 133 (49.8%) in CG. After 52 weeks (T3), data from 62.7% (*n* = 173/267) were evaluable, the active dropout rate (medical reasons or self-request) was 19.1% (IG: *n* = 32; CG: *n* = 19) (Fig. 1). The remaining 92 CAYAs without a high need for dietary intervention are included in Table 1 for group comparison.

**Table 1:** Patient characteristics of the CFC-nutrition module compared to remaining patients with no high need for the CFC-nutrition module

	% (No.)	
	Total RCT cohort	
	***RCT cohort CFC-nutrition module***	***Remaining patients with no high need for the CFC-nutrition module***
Demographic data		
Total participants (*n*)	267	92
Female	66.7% (178)	51.1% (47)
Male	33.3% (89)	48.9% (45)
Age, median (IQR), y	24.8 (19.6; 31.6)	27.7 (20.7; 33.4)
Highest school degree		
Total participants (*n*)	228	85
Baccalaureate	48.7% (111)	49.4% (42)
Advanced technical college	13.6% (31)	11.8% (10)
Secondary school leaving certificate	32.5% (74)	35.3% (30)
Elementary or secondary school diploma	4.8% (11)	2.4% (2)
Other	0.4% (1)	1.2% (1)
No school diploma	0.0% (0)	3.5% (3)
Employment status		
Total participants (*n*)	246	82
Student, school/university	38.2% (94)	32.9% (27)
Employed, full/part time	48.8% (120)	57.3% (47)
Unemployed	8.1% (20)	4.9% (4)
Early retirement/disability pensioners	4.9% (12)	4.9% (4)
Anthropometric data, median (IQR)		
Height (m)	1.69 (1.64; 1.77)	1.74 (1.68; 1.79)
Weight (kg)	68.4 (58.0; 83.0)	67.0 (60.5; 75.0)
BMI (kg/m^2^)	23.8 (20.8; 27.5)	22.4 (20.8; 24.9)
WHR	0.83 (0.77; 0.89)	0.81 (0.76; 0.86)
Tumor specific data		
Total participants (*n*)	199	82
Lymphoma	32.5% (65)	35.4% (29)
Sarcoma	15.5% (31)	17.1% (14)
Leukemia	12.0% (24)	19.5% (16)
Brain tumor	16.0% (32)	3.7% (3)
Thyroid carcinoma	4.5% (9)	0% (0)
Urological tumor	6.0% (12)	11.0% (9)
Gynecological tumor	7.5% (15)	4.9% (4)
Other solid tumors	2.5% (5)	2.4% (2)
Gastrointestinal tumor	1.5% (2)	2.4% (2)
Head and neck tumor	1.0% (2)	3.7% (3)
Skin tumor	1.0% (2)	0% (0)
Recurrence or new disease	16.9% (32/189)	22.0% (15/68)
Months since therapy, median (IQR)	68.0 (17.5; 127.5)	47.0 (13.0; 109.0)

*IG* intervention group,* CG* control group,* IQR* interquartile range,* m* meter,* kg* kilogram,* BMI* body mass index, *WHR* waist-hip ratio,* y* year,* No.* number; *n* = sample size

### Patient characteristics

Most participants with a high need for the CFC-nutrition module had an additional high need in the module psycho-oncology (70.0%, *n* = 187) and physical activity (70.4%, *n* = 188). Only 9.0% (*n* = 24) reported an isolated high need in the CFC-nutrition module. The remaining participants with no high need for the CFC-nutrition module had a shorter time since completion of therapy, were less likely to be female, and had a slightly lower BMI (Table 1). Patient characteristics between IG and CG were well balanced and shown in Table A4.

### Adherence to the program and further informations

The adherence to the CFC-nutrition module (attending all five counseling sessions) was 59.6% (*n* = 65) in the IG and the one-time counseling in the CG took place in 70.6% (*n* = 89) [10]. About 63.6% (*n* = 84) participate in the grocery shopping training and 67.4% (*n* = 89) in the cooking class*.* About 41.7% (*n* = 82) received dietary counseling before the CFC-P, during, or after cancer therapy. Only 24.6% (*n* = 49) reported nutritional counseling during their cancer aftercare. Regarding the taste perception, descriptive data indicated that 59 of 267 participants reported problems: 55.9% (*n* = 33) had taste impairments, 30.5% (*n* = 18) had smell impairments, and 13.6% (*n* = 8) had both. Of 54 participants of the Hamburg subgroup, about 9.3% (*n* = 5) had taste disorder (≤ 8 points; “taste strips”). Regarding the defining criteria of the “high need,” both groups have shown similar findings (Table A5).

**Fig. 2:**
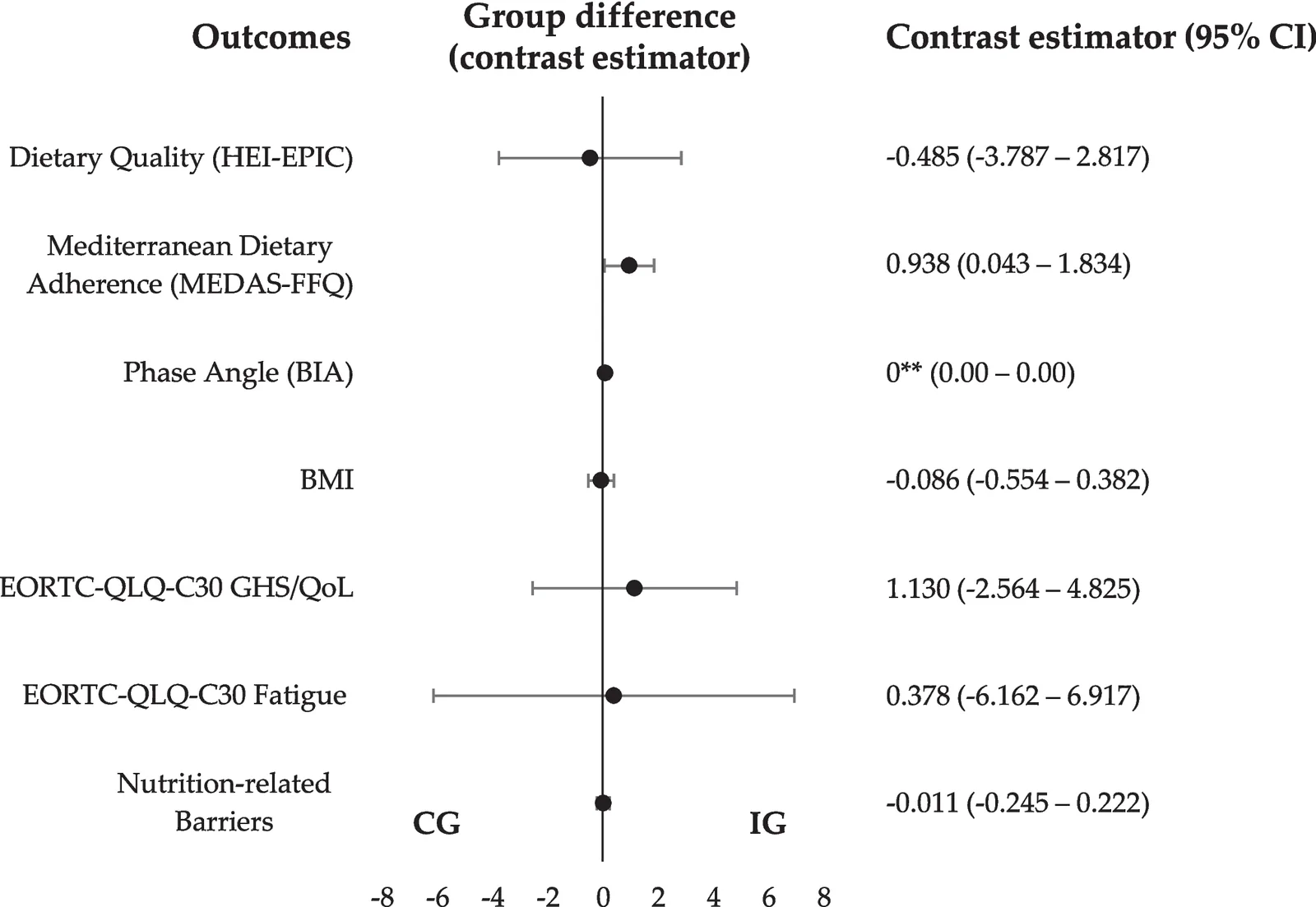
Forest plot of group difference (contrast estimates) with 95% CI across all outcomes

Figure 2 shows a forest plot of model-estimated group differences (contrast estimates) with 95% CI within the Intention-to-Treat (ITT) population for all outcomes. Positive values indicate outcomes favorable to the IG, while negative values indicate outcomes favorable to the CG. All outcomes are described in more detail below. Similar results were obtained in the Per-Protocol (PP) population.

### Dietary quality: changes of the dietary quality and Mediterranean dietary adherence

Regarding the dietary quality, no significant difference could be detected between IG and CG at T3 (contrast estimate, −0.485; 95% CI, −3.787 to 2.817; *p* = 0.773). However, in both groups, an improvement in dietary quality was observed over the time from T1 to T3 (Table 2).

**Table 2:** Dietary quality and Mediterranean dietary adherence

Intra-group comparison (median)*							Group comparison (mean)	
	IG			CG				
Outcome	***T*** (***n*** =)	Median (IQR)	Diff. T1	***T*** (***n*** =)	Median (IQR)	Diff. T1	Contrast estimator (95% CI)	***p***-value
Dietary quality—HEI-EPIC (score)	1 (127) 2 (93) 3 (87)	50.0 (42.0; 63.0) 56.0 (49.0; 65.0) 58.0 (43.0; 71.0)	- ^a^ + 4.0 ^b^ + 2.0 ^b^	1 (130) 2 (95) 3 (86)	49.0 (39.0; 62.0) 52.0 (41.0; 68.0) 57.5 (47.0; 68.0)	- ^a^ + 2.0 ^a^ + 2.0 ^b^	2.773 (−0.425–5.971) −0.485 (−3.787–2.817)	0.089 0.773
Mediterranean dietary adherence—MEDAS-FFQ (score)	1 (75) 2 (57) 3 (49)	6.0 (4.0; 7.0) 7.0 (6.0; 8.0) 7.0 (6.0; 8.0)	- ^a^ + 1.0 ^b^ + 2.0 ^b^	1 (61) 2 (47) 3 (48)	6.0 (4.0; 8.0) 7.0 (5.0; 9.0) 7.0 (5.0; 9.0)	- ^a^ + 0.0 ^a^ + 1.0 ^a^	0.560 (−0.357–1.477) 0.938 (0.043–1.834)	0.228 0.040

^a,b^Medians followed by the same letter are not significantly different at alpha = 0.05 (not adjusted for multiple testing)

*IG* intervention group, *CG* control group, *IQR* interquartile range, *CI* confidence interval, *T* time point of measurement, *Diff.* difference; *n* = sample size

A statistically significant improvement was seen in the IG from T1 to T2 (*p* < 0.0001) and T1 to T3 (*p* = 0.028), and in the CG from T1 to T3 (*p* = 0.009) (Table A6). A good, moderate, and poor dietary quality (HEI-EPIC categories) was seen in the IG in 11.0% (*n* = 14/128), 61.7% (*n* = 79/128), and 27.3% (*n* = 35/128) at T1 and in 15.6% (*n* = 14/90), 63.3% (*n* = 57/90), and 21.1% (*n* = 19/90) at T3. In the CG, 11.5% (*n* = 15/131) had a good, 54.2% (*n* = 71/131) a moderate, and 34.3% (*n* = 45/131) a poor dietary quality at T1, while 15.1% (*n* = 13/86), 68.6% (*n* = 59/86), and 16.3% (*n* = 14/86) at T3, respectively. CAYAs (both groups) had a moderate dietary quality at all time points (based on the median). Between the IG and CG was a significant change for the food group “meat, sausage, fish, egg, and plant-based derivatives” at T3 (*p* = 0.043). Medians are shown in Fig. A1. A sensitivity analysis of CAYAs with an isolated high need in the CFC-nutrition module (*n* = 24) showed median improvements of 7.0 pts (T2) and 5.0 pts (T3) in the IG and 0.0 pts (T2) and 1.0 pts (T3) in the CG, with no significant improvement at T3 (*p* = 0.957). Regarding the Mediterranean dietary adherence*,* significant improvements for IG at T3 could be observed (contrast estimate, 0.938; 95% CI, 0.043 to 1.834; *p* = 0.040). The median score improved in both groups (Table 2 and Table A6). CAYAs in both groups demonstrated a moderate adherence to a Mediterranean dietary pattern at all measurement time points. In the IG, high, moderate, and low adherence to a Mediterranean diet was observed at T1 in 4.0% (*n* = 3/75), 53.3% (*n* = 40/75), and 42.7% (*n* = 32/75) of CAYAs. Correspondingly, at T3, the adherence amounted to 14.6% (*n* = 7/48), 68.7% (*n* = 33/48), and 16.7% (*n* = 8/48) of CAYAs. In the CG, 3.3% (*n* = 2/60) had a high adherence, 56.7% (*n* = 34/60) had a moderate adherence, and 40.0% (*n* = 24/60) had a low adherence to the Mediterranean diet at T1, while 23.4% (*n* = 11/47), 42.6% (*n* = 20/47), and 34.0% (*n* = 16/47) had these levels of adherence at T3.

### Physical outcomes: phase angle (bioelectrical impedance analysis (BIA)) and body mass index (BMI)

Regarding the phase angle, no significant changes between IG and CG at T3 (*p* = 0.937) could be found (Table 3). At T3, 34.0% (*n* = 18) of the IG and 29.6% (*n* = 16) of the CG group improved their phase angle by 5%.

**Table 3:** Physical outcomes

Intra-group comparison (median)*							Group comparison (mean)	
	IG			CG				
Outcome	***T*** (***n*** =)	Median (IQR)	Diff. T1	***T*** (***n*** =)	Median (IQR)	Diff. T1	Contrast estimator (95% CI)	***p***-value
Phase angle—BIA (°)	1 (94) 2 (79) 3 (59)	6.0 (5.4; 6.5) 5.9 (5.5; 6.6) 5.9 (5.6; 6.6)	- ^a^ + 0.1 ^a^ + 0.1 ^a^	1 (88) 2 (63) 3 (62)	5.9 (5.4; 6.5) 6.1 (5.4; 6.3) 6.1 (5.6; 6.4)	- ^a^ + 0.0 ^a^ + 0.1 ^a^	0.059 (−0.074–0.191) 0** (0.00–0.00)	0.382 0.937
Body mass index (BMI)—(kg/m^2^)	1 (131) 2 (70) 3 (87)	24.3 (21.8; 28.7) 24.1 (20.9; 27.4) 24.3 (21.6; 29.0)	- ^a^ - 1.0 ^a^ + 0.0 ^a^	1 (131) 2 (58) 3 (88)	23.3 (20.0; 26.0) 24.6 (22.0; 29.6) 23.8 (21.2; 26.6)	- ^a^ + 0.0 ^a^ + 1.0 ^a^	−0.284 (−0.673–0.104) −0.086 (−0.554–0.382)	0.150 0.717
Coefficient is set to 0 as redundant								

^a,b^Medians followed by the same letter are not significantly different at alpha = 0.05 (not adjusted for multiple testing)

*IG* intervention group,* CG* control group,* IQR* interquartile range,* CI* confidence interval,* T* time point of measurement,* Diff.* difference; *n* = sample size

No significant changes could be found in the different BMI groups between IG and CG at T3 (*p* = 0.717). The rate of CAYAs with underweight was stable in both groups, ranging from 9.0% (*n* = 19/210) (T1) to 9.8% (*n* = 12/123) (T3). Overweight increased in both groups from 21.0% (*n* = 44/210) (T1) to 22.0% (*n* = 27/123) (T3), and obesity (grade 1–3) increased from 20.0% (*n* = 42/210) (T1) to 22.0% (*n* = 27/123) (T3). Median BMI did not change in the IG, while in the CG, the BMI increased by 0.5 kg/m^2^ (Table 3).

### Mental outcomes: EORTC-QLQ-C30 Global Health Score (GHS)/quality of life (QoL), fatigue, nutrition-related barriers, and satisfaction with the program

The EORTC-QLQ-C30 GHS/QoL increased with 7.1 pts (IG) and 5.4 pts (CG) (contrast estimate, 1.130; 95% CI, −2.564 to 4.825; *p* = 0.547) (Table 4). The increase from T1 to T2 and from T1 to T3 in the IG and in the CG (*p* < 0.0001) was statistically significant (Table A6). Neither group reached the 10-point threshold of improvement in terms of clinical relevance.

**Table 4:** Mental outcomes

Intra-group comparison (median)*								Group comparison (mean)	
	IG				CG				
Outcome	***T*** (***n*** =)	Median (IQR)		Diff. T1	***T*** (***n*** =)	Median (IQR)	Diff T1	Contrast estimator (95% CI)	***p***-value
EORTC-QLQ-C30 GHS/QoL (score)	1 (132) 2 (108) 3 (98)	73.7 (61.1; 83.4) 78.4 (67.7; 91.4) 82.1 (67.7; 92.4)	- ^a^ + 5.7 ^b^ + 7.1 ^b^		1 (132) 2 (104) 3 (95)	78.9 (63.7; 88.6) 85.0 (72.5; 92.3) 86.5 (75.3; 96.1)	- ^a^ + 5.1 ^b^ + 5.4 ^b^	1.410 (−1.579–4.399) 1.130 (−2.564–4.825)	0.354 0.547
EORTC-QLQ-C30 Fatigue (score)	1 (132) 2 (110) 3 (99)	55.6 (33.3; 72.2) 44.4 (22.2; 66.7) 44.4 (22.2; 66.7)	- ^a^ - 11.1 ^a^ - 11.1 ^a^		1 (132) 2 (104) 3 (96)	44.4 (33.3; 66.7) 33.3 (22.2; 55.6) 33.3 (22.2; 47.2)	- ^a^ - 11.1 ^a^ - 11.1 ^a^	−0.446 (−6.735–5.843) 0.378 (−6.162–6.917)	0.889 0.910
Nutrition-Related Barriers (total score)	1 (102) 2 (95) 3 (82)	3.0 (2.6; 3.4) 2.9 (2.4; 3.4) 2.8 (2.3; 3.3)	- ^a^ - 0.1 ^a^ - 0.1 ^a^		1 (95) 2 (78) 3 (76)	3.0 (2.5; 3.4) 3.0 (2.6; 3.3) 2.9 (2.5; 3.4)	- ^a^ - 0.1 ^a^ - 0.1 ^a^	−0.055 (−0.298–0.187) −0.011 (−0.245–0.222)	0.646 0.923

^a,b^Medians followed by the same letter are not significant different at alpha = 0.05 (not adjusted for multiple testing)

*IG* intervention group,* CG* control group,* IQR* interquartile range,* CI* confidence interval,* T* time point of measurement,* Diff.* difference; *n* = sample size

About 94.3% (*n* = 249) experienced at least mild fatigue (> 10 points). The EORTC-QLQ-C30 Fatigue score decreased in the IG and in the CG by 11.1 points at T3 (*p* = 0.910) (Table 4). The improvement from T1 to T2 and from T1 to T3 in the IG and in the CG (*p* < 0.0001) was statistically significant (Table A6). Both groups reached the 10-point threshold of improvement for fatigue in terms of clinical relevance.

Regarding the nutrition-related barriers (see Fig. A2 for more details), no significant difference could be detected between the IG and the CG at T3 (*p* = 0.923). TTM-stages (see Fig. A3 for more details) could be improved, but with no significant difference between the groups at T3 (*p* = 0.454).

Overall, 89.7% were satisfied (highest or second highest response) with the CFC-nutrition module (IG: 92.6%; CG: 85.4%), while 64.3% of the IG and 35.1% of the CG gave of the highest possible response (Fig. A4).

## Discussion

To our knowledge, there has been no prospective dietary intervention in a multicenter lifestyle follow-up care program for young cancer survivors so far. Around 75% of CAYAs reported a high need for the CFC-nutrition module, corroborating the high relevance of our study. This need was almost identical to the need for psychosocial support, which was expected [3] and confirmed in the CFC-P to be as high as roughly 75% [10].

The intervention of the CFC-nutrition module slightly improved dietary quality (HEI-EPIC) in both groups and the Mediterranean dietary adherence (MEDAS-FFQ) significantly improved in the IG. Empowerment to make lifestyle/nutrition-related behavioral changes (TTM) was increased and participants showed a high satisfaction with the dietary intervention, more so in the IG. Physical outcomes such as BMI or phase angle (BIA) did not improve.

### Dietary outcomes

Other dietary interventional studies in cancer survivors have shown that dietary interventions have a moderate positive effect on dietary quality and on the fruit and vegetable intake [39]. Participation in the module showed clear effects on dietary outcomes, although the intensive approach in the IG led only to a slight overall improvement. The multimodal approach may have led to an overlapping of needs and an intervention overload. In addition, the similar results in both groups allow for assuming that one counseling session may in fact be sufficient. Two important aspects to consider are that the primary outcome measurement period of 52 weeks may have been too long and that the “HEI-EPIC” may not have been specific enough for outcome measurement. In addition, simply keeping a personal food diary was observed to encourage reflection on eating habits and prompt changes [40]. This could suggest that by simply raising dietary awareness through counseling (e.g., by at least one counseling session for young cancer survivors) is, to a lesser or greater extent, more beneficial than having no counseling at all.

The rate of active dropout and lost follow-ups in the CFC-nutrition module was around 37.3%, which is comparable to other dropout rates in CAYAs [41]. The main barriers to long-term follow-up care were a lack of resources, information, and awareness of care [42]. These issues should be considered when designing future CAYA programs.

Due to the multimodal approach of CFC-P and the small sample size (*n* = 24), the effectiveness of an isolated dietary intervention could not be analyzed. It must also be considered that CAYAs of CFC-module nutrition are a heterogeneous group and vary in many characteristics: long-term effects, tumor entities/therapies, nutritional problems, or, for example, in their employment status (around 50% employees; 35% students).

Measuring dietary quality remains challenging [43] and can be subject to many biases [44]. Validated tools remain limited [19], the HEI-EPIC has limitations such as the lack of differentiation between specific food groups [29], and dietary recording is prone to errors such as over-reporting [45].

Completing a dietary record directly reflects dietary quality and may have had an influence in terms of “reactivity bias” [44]. This may have increased the awareness of better self-care in the CG as well.

Cancer survivors, such as up to 2/3 of breast, colorectal, and prostate cancer survivors [46], are more motivated to make lifestyle changes subsequent to the diagnosis [47]. It remains difficult to change dietary behavior of cancer survivors and/or to make this visible in studies [39]. Overall, our intervention could not improve significantly the dietary quality in total. However, we saw improvements regarding the Mediterranean dietary adherence, which is broadly in line with healthy eating recommendations [48]. The heart-healthy Mediterranean diet takes on particular relevance given the increased cardiovascular risk among young cancer survivors, who are seven times more likely to develop cardiovascular disease than the general population [49]. Finally, the participants’ compliance and effects of our intervention are limited to specific aspects, but well suited to real-world conditions where multiple lifestyle factors have to be addressed [50].

### Mental outcomes

As a multimodal program, CFC-P aimed to also improve mental health and empowerment/self-motivation strategies. Over 90% of the CAYAs in the module had at least mild fatigue consistent with preceding studies [51]. Our baseline results for EORTC-QLQ-C30 Fatigue and GHS/QoL are higher compared to the average score of the EORTC-QLQ-C30 cohort [52]. However, comparability with patients undergoing treatment remains limited; for this purpose, the EORTC SURV-100 questionnaire was developed [53]. Although the differences between the groups were not statistically significant, both groups showed a median improvement in fatigue of 11 points, which exceeds the established Minimal Clinically Important Difference (MCID) threshold of 10 points and therefore suggests clinical relevance. EORTC-QLQ-C30 GHS/QoL also improved in both groups, even if it did not reach the 10-point threshold. Our data are in line with several trials of dietary interventions, often combined with physical activity, which have also shown improvements in QoL [54] and fatigue [55] in cancer survivors.

The use of behavior change strategies was successfully implemented in our module and in line with recommendations for cancer survivors [39]. Empowerment for lifestyle/nutritional behavior changes was also improved (TTM-Stages), which may indicate a better dietary quality [56].

### Implications

A dietary intervention for cancer survivors is recommended by the World Cancer Research Fund (WCRF) [57]. However, only around 25% of CAYAs received counseling before the CFC-P, stressing the need of structured survivorship programs including nutritional counseling.

Intentional dietary behavior change is a complex and sequential phenomenon [58]. Long-term consequences of unhealthy lifestyles associated with treatment are often not amenable to simple habit change—but a health promotion conversation can facilitate health behavior change in CAYAs [59]. Our dietary needs assessment and personalized counseling could be transferred to other survivorship programs. Based on this, we are currently conducting precise dietary intervention studies for long-term effects (e.g., Gut Health; Register-ID: DRKS00025802).

Our findings demonstrate the intervention’s feasibility. Around 75% of the CAYAs had high dietary needs and the need-stratified assessment proved sufficient. Due to reduced reliability of the HEI-EPIC (also feedback from other study centers), a screening tool (Short Healthy Eating Index Hamburg; Register-ID: DRKS00032249) was developed and used after RCT (will be published elsewhere). For young cancer survivors, personalized nutritional advice remains an important part of follow-up care.

## Conclusions

Structured assessment of dietary quality and individualized interventions could provide ideal supportive care in survivorship programs. Our screening tool(s) used and developed could be introduced in the context of long-term oncological follow-up.

A high need for dietary intervention was identified in three out of four CAYAs. The dietary intervention in the CFC-P did not lead to significant changes in the dietary quality (primary endpoint) between the groups, but it did result in significant differences in the Mediterranean diet adherence between the groups, and the intervention was feasible for young cancer survivors. However, it should be noted that the analyses were exploratory in nature. Furthermore, there was an improvement in mental health, a high level of satisfaction with the intervention, and an empowerment for the lifestyle/nutritional behavior change among participants of the CFC-nutrition module.

## Supplementary Information

Below is the link to the electronic supplementary material.

ESM 1 (DOCX 77.9 KB)

ESM 2 (DOCX 432 KB)

## Data Availability

De-identified data from this study are not available in a public archive. De-identified data from this study will be made available (as allowable according to Institutional Review Board (IRB) standards) by emailing the corresponding author.
