# Supplementary material for: Dietary intervention among young cancer survivors within the CARE for CAYA program
Source: J Cancer Surviv. 2026 Mar 26;20(Suppl 2):1754–66. doi: 10.1007/s11764-026-01997-4 (PMC13744525; doi:10.1007/s11764-026-01997-4)
Supplement: Supplementary file 1 [file 11764_2026_1997_MOESM1_ESM.docx]

**Appendix A: Supplementary materials**: Tables and Text

**Table A1.** Reporting checklist for randomized trial based on the CONSORT guidelines [1].

|  | **Section/topic** | **No** | **CONSORT 2025 checklist item description** | **Reported on page no.** |
| --- | --- | --- | --- | --- |
|  | **Title and abstract** | | |  |
|  | Title and structured abstract | 1a | Identification as a randomized trial | 5 |
|  |  | 1b | Structured summary of the trial design, methods, results, and conclusions | 5 |
|  | **Open science** | | |  |
|  | Trial registration | 2 | Name of trial registry, identifying number (with URL) and date of registration | 5 |
|  | Protocol and statistical analysis plan | 3 | Where the trial protocol and statistical analysis plan can be accessed | 27 |
|  | Data sharing | 4 | Where and how the individual de-identified participant data (including data dictionary), statistical code and any other materials can be accessed | 27 |
|  | Funding and conflicts of interest | 5a | Sources of funding and other support (eg, supply of drugs), and role of funders in the design, conduct, analysis and reporting of the trial | 27 |
|  |  | 5b | Financial and other conflicts of interest of the manuscript authors | 27-28 |
|  | **Introduction** | | |  |
|  | Background and rationale | 6 | Scientific background and rationale | 7-8 |
|  | Objectives | 7 | Specific objectives related to benefits and harms | 7-8 |
|  | **Methods** | | |  |
|  | Patient and public involvement | 8 | Details of patient or public involvement in the design, conduct and reporting of the trial | - |
|  | Trial design | 9 | Description of trial design including type of trial (eg, parallel group, crossover), allocation ratio, and framework (eg, superiority, equivalence, non-inferiority, exploratory) | 9 |
|  | Changes to trial protocol | 10 | Important changes to the trial after it commenced including any outcomes or analyses that were not prespecified, with reason | - |
|  | Trial setting | 11 | Settings (eg, community, hospital) and locations (eg, countries, sites) where the trial was conducted | 9 |
|  | Eligibility criteria | 12a | Eligibility criteria for participants | 9 |
|  |  | 12b | If applicable, eligibility criteria for sites and for individuals delivering the interventions (eg, surgeons, physiotherapists) | 9 |
|  | Intervention and comparator | 13 | Intervention and comparator with sufficient details to allow replication. If relevant, where additional materials describing the intervention and comparator (eg, intervention manual) can be accessed | 9-10 |
|  | Outcomes | 14 | Prespecified primary and secondary outcomes, including the specific measurement variable (eg, systolic blood pressure), analysis metric (eg, change from baseline, final value, time to event), method of aggregation (eg, median, proportion), and time point for each outcome | 10 |
|  | Harms | 15 | How harms were defined and assessed (eg, systematically, non-systematically) | 9 |
|  | Sample size | 16a | How sample size was determined, including all assumptions supporting the sample size calculation | 11 |
|  |  | 16b | Explanation of any interim analyses and stopping guidelines | - |
|  | Randomisation: |  |  |  |
|  | Sequence generation | 17a | Who generated the random allocation sequence and the method used | 9 |
|  |  | 17b | Type of randomisation and details of any restriction (eg, stratification, blocking and block size) | 9 |
|  |  |  |  | **Reported on page no.** |
|  | Allocation concealment mechanism | 18 | Mechanism used to implement the random allocation sequence (eg, central computer/telephone; sequentially numbered, opaque, sealed containers), describing any steps to conceal the sequence until interventions were assigned | 9 |
|  | Implementation | 19 | Whether the personnel who enrolled and those who assigned participants to the interventions had access to the random allocation sequence | 9 |
|  | Blinding | 20a | Who was blinded after assignment to interventions (eg, participants, care providers, outcome assessors, data analysts) | 9 |
|  |  | 20b | If blinded, how blinding was achieved and description of the similarity of interventions | - |
|  | Statistical methods | 21a | Statistical methods used to compare groups for primary and secondary outcomes, including harms | 11 |
|  |  | 21b | Definition of who is included in each analysis (eg, all randomized participants), and in which group | 11 |
|  |  | 21c | How missing data were handled in the analysis | 11 |
|  |  | 21d | Methods for any additional analyses (eg, subgroup and sensitivity analyses), distinguishing prespecified from post hoc | 11 |
|  | **Results** | | |  |
|  | Participant flow, including flow diagram | 22a | For each group, the numbers of participants who were randomly assigned, received intended intervention, and were analyzed for the primary outcome | 11-12 & 15-16 |
|  |  | 22b | For each group, losses and exclusions after randomization, together with reasons | 11-12 |
|  | Recruitment | 23a | Dates defining the periods of recruitment and follow-up for outcomes of benefits and harms | 11-12 |
|  |  | 23b | If relevant, why the trial ended or was stopped | - |
|  | Intervention and comparator delivery | 24a | Intervention and comparator as they were actually administered (eg, where appropriate, who delivered the intervention/comparator, how participants adhered, whether they were delivered as intended (fidelity)) | 11-12 |
|  |  | 24b | Concomitant care received during the trial for each group | 9-10 |
|  | Baseline data | 25 | A table showing baseline demographic and clinical characteristics for each group | 12-13 |
|  | Numbers analysed,  outcomes and estimation | 26 | For each primary and secondary outcome, by group:  ● the number of participants included in the analysis  ● the number of participants with available data at the outcome time point  ● result for each group, and the estimated effect size and its precision (such as 95% confidence interval)  ● for binary outcomes, presentation of both absolute and relative effect size | 15-18 |
|  | Harms | 27 | All harms or unintended events in each group | 15 |
|  | Ancillary analyses | 28 | Any other analyses performed, including subgroup and sensitivity analyses, distinguishing pre-specified from post hoc | 14 |
|  | **Discussion** | | |  |
|  | Interpretation | 29 | Interpretation consistent with results, balancing benefits and harms, and considering other relevant evidence | 18-21 |
|  | Limitations | 30 | Trial limitations, addressing sources of potential bias, imprecision, generalizability, and, if relevant, multiplicity of analyses | 18-21 |

**Table A2.** Tidier-Checklist [2].

| **Item number** | **Item** | **Where located **** | |
| --- | --- | --- | --- |
|  |  | Primary paper  (page or appendix  number) | Other ^†^ (details) |
|  | **BRIEF NAME** |  |  |
| **1.** | Provide the name or a phrase that describes the intervention. | ___9-10______ | ______________ |
|  | **WHY** |  |  |
| **2.** | Describe any rationale, theory, or goal of the elements essential to the intervention. | ____7-8______ | _____________ |
|  | **WHAT** |  |  |
| **3.** | Materials: Describe any physical or informational materials used in the intervention, including those provided to participants or used in intervention delivery or in training of intervention providers. Provide information on where the materials can be accessed (e.g. online appendix, URL). | ____9-10_____ | _____________ |
| **4.** | Procedures: Describe each of the procedures, activities, and/or processes used in the intervention, including any enabling or support activities. | _____9-10____ | _____________ |
|  | **WHO PROVIDED** |  |  |
| **5.** | For each category of intervention provider (e.g. psychologist, nursing assistant), describe their expertise, background and any specific training given. | ______10______ | _____________ |
|  | **HOW** |  |  |
| **6.** | Describe the modes of delivery (e.g. face-to-face or by some other mechanism, such as internet or telephone) of the intervention and whether it was provided individually or in a group. | ______9-10___ | _____________ |
|  | **WHERE** |  |  |
| **7.** | Describe the type(s) of location(s) where the intervention occurred, including any necessary infrastructure or relevant features. | _______9-10__ | _____________ |
|  | **WHEN and HOW MUCH** |  |  |
| **8.** | Describe the number of times the intervention was delivered and over what period of time including the number of sessions, their schedule, and their duration, intensity or dose. | ____9-10_____ | _____________ |
|  | **TAILORING** |  |  |
| **9.** | If the intervention was planned to be personalised, titrated or adapted, then describe what, why, when, and how. | _____-_______ | _____________ |
|  | **MODIFICATIONS** |  |  |
| **10.^ǂ^** | If the intervention was modified during the course of the study, describe the changes (what, why, when, and how). | ______-______ | _____________ |
|  | **HOW WELL** |  |  |
| **11.** | Planned: If intervention adherence or fidelity was assessed, describe how and by whom, and if any strategies were used to maintain or improve fidelity, describe them. | ______-______ | _____________ |
| **12.^ǂ^** | Actual: If intervention adherence or fidelity was assessed, describe the extent to which the intervention was delivered as planned. | _____-_______ | _____________ |

**Table A3.** Outcomes of the Module CFC-Nutrition.

| Outcomes | Description | Data Assessment |
| --- | --- | --- |
| Primary outcome | **Dietary Quality**  *Improvement in dietary quality compared to baseline measurement* | *Healthy Eating Index (HEI-EPIC Score)* [3, 4]  The HEI is a tool for assessing the dietary quality [5], the validated German version was used [4, 6]. The HEI-EPIC was formerly used in the INAYA trial and was considered as appropriate for CAYAs [7]. To calculate the HEI-EPIC, based on eight food groups, participants filled out a three-day dietary record (“Freiburger Ernährungsprotokoll”) [8], which was analyzed by the CFC-P nutritionists and dieticians using an Excel tool developed by ©Julia von Grundherr. This analysis is based on a ratio in points between recommended and consumed portions, taking into account the recommendation of the German Nutrition Society (Deutsche Gesellschaft für Ernährung (DGE)) [9]. The range for each food group was 0-10 points. In fruit, vegetables and beverages groups up to 20 points [3]. This resulted in a score range of up to 110 points, with higher scores indicating a better dietary quality [3]. This score allowed to categorize the dietary quality as following: ≤ 40 points indicate a poor, 41-64 points a moderate and ≥ 65 points a good dietary quality. |
| Secondary outcomes | **Phase Angle**  *Improvement of the phase angle by 5% compared to the baseline measurement* | *Bioelectrical Impedance Analysis (BIA)*  BIA is a technique for assessing body composition and to providing the phase angle [10]. A decreased phase angle is considered a marker of inflammatory disease [11], a prognostic marker for morbidity and mortality in chronic inflammatory conditions [12] and obesity [13]. Increased phase angle is an indicator of cellular health [14, 15]. |
|  | **Mediterranean Dietary Adherence** *Improvement of the Mediterranean dietary adherence* | *Mediterranen Dietary Behavior Questionnaire (MEDAS-FFQ)* [16]  The MEDAS-FFQ included 14 questions about the consumption of food groups and eating habits typical of the Mediterranean diet [17]. The scoring for question 8 was slightly changed (one point was given for "less than 1 glass"). This change is based on recommendations from the World Cancer Research Fund (WCRF) because alcohol increases the risk of cancer [18]. A score of 14 points was possible, with higher scores indicating a better Mediterranean dietary quality [16]. An adherence could be categorized as low (≤ 5 points), moderate (6-9 points), and high (≥10 points). |
|  | **Nutrition-related Barriers**  *Reduction of nutrition-related barriers* | *Transtheoretical Model (TTM) - Step Algorithm for Nutrition* [19]  *Decision balance short form (modified)* [19]  TTM describes the stages of goal-directed behavior change [20] and is divided into five stages. This could be assessed in the validated TTM – Step Algorithm for Nutrition, within three questions and can applied to dietary changes [19]. The level of motivation of the patients can be applied to the respective stage and the counseling can be tailored accordingly [21]. The questions "Do you eat a healthy diet most of the time, i.e. a varied diet with a high proportion of plant-based foods? " have been slightly reworded, but without changing the meaning. Additionally, the *Decision Balance Short Form (modified)* was used. For the question "If I eat a healthy diet, i.e. a varied diet with a high proportion of plant foods..." a five-point scale for five barriers and five facilitators was used. The questions have been slightly reworded. Based on an algorithm by Keller et al, the two questionnaires can be evaluated and the correct TTM stage could be identified [19]. |
|  | **Body Mass Index (BMI)**  *Decrease in the number of patients who are underweight, overweight, or obese by BMI compared to baseline measurement* | *BMI (WHO)* [22] |
|  | **EORTC-QLQ-C30 Global Health Score (GHS) and Fatigue Score**  *Improvement of global health score/quality of life and fatigue* | *European Organization for Research and Treatment of Cancer QLQ-C30 (EORTC QLQ-C30)* [23]  Questions 29 and 30 to assess the global health score in terms of quality of life. The fatigue subscore of the EORTC-C30 was scored according to the formula of Fayers et al. [24]; based on guidelines and on prior assumptions, fatigue can be categorized as none (< 10 points), mild (10- 40 points), moderate (40-< 70 points), and severe (≥ 70 points) [25-27]. A score of 0 to 100 points is possible, with higher scores indicating a better quality of life [24]. Higher scores correspond to more severe fatigue symptoms [24]. |
|  | **Subjective And Objective Taste Perception**  *Improvement of subjective and objective taste perception (subgroup at centers)* | *Taste test* "*taste strips*" [28]  *Questionnaire by T. Hummel* [29]  The questionnaire by T. Hummel was only filled out in case of problems indicated, and the validated taste test "taste strips" was assessed in the subgroup of participants at the site UKE Hamburg. The taste strip test based on patients' responses, a taste score (0-16 points) was used to identify taste dysfunction (≤8 points) and no taste dysfunction (≥9 points) [28]. |

**Table A4.** Patient Characteristics of the Module CFC-Nutrition (IG and CG).

| **% (No.)** | | | | |  | |  |
| --- | --- | --- | --- | --- | --- | --- | --- |
| ***RCT Cohort CFC-nutrition module*** | | | | | | | |
|  | | | ***IG*** | | | ***CG*** | |
| **Demographic data** | | | |  |  |  |  |
| Total Participants (n) | | | 134 | | | 133 | |
| Female | | | 63.4% (85) | | | 69.9% (93) | |
| Male | | | 36.6% (49) | | | 30.1% (40) | |
| Age, median (IQR), y | | | 24.8 (19.8; 31.6) | | | 24.4 (19.6; 32.2) | |
| **Highest school degree** |  | | |  |  |  |  |
| Total Participants (n) | | | 115 | | | 113 | |
| Baccalaureate | | | 46.1% (53) | | | 51.3% (58) | |
| Advanced technical college | | | 15.7% (18) | | | 11.5% (13) | |
| Secondary school leaving certificate | | | 32.2% (37) | | | 32.7% (37) | |
| Elementary or secondary school diploma | | | 5.2% (6) | | | 4.4% (5) | |
| Other | | | 0.9% (1) | | | 0.0% (0) | |
| No school diploma | | | 0.0% (0) | | | 0.0% (0) | |
| **Employment status** |  | | |  |  |  |  |
| Total Participants (n) | | | 123 | | | 123 | |
| Student, school/university | | | 35.0% (43) | | | 41.5% (51) | |
| Employed, full/part time | | | 50.4% (62) | | | 47.2% (58) | |
| Unemployed | | | 8.1% (10) | | | 8.1% (10) | |
| Early retirement/disability pensioners | | | 6.5% (8) | | | 3.3% (4) | |
| **Anthropometric data, median (IQR)** | |  |  |  |  |  |  |
| Height [m] | | | 1.69 (1.64; 1.78) | | | 1.68 (1.62; 1.76) | |
| Weight [kg] | | | 73.0 (61.5; 85.8) | | | 65.0 (56.0; 78.0) | |
| BMI [kg/m^2^] | | | 24.5 (21.8; 28.7) | | | 23.3 (20.0; 25.9) | |
| WHR | | | 0.83 (0.77; 0.89) | | | 0.82 (0.78; 0.89) | |
| **Tumor specific data** | | | |  |  |  |  |
| Total Participants (n) | | | 95 | | | 104 | |
| Lymphoma | | | 34.4% (33) | | | 30.8% (32) | |
| Sarcoma | | | 13.5% (13) | | | 17.3% (18) | |
| Leukemia | | | 13.5% (13) | | | 10.6% (11) | |
| Brain tumor | | | 12.5% (12) | | | 19.2% (20) | |
| Thyroid carcinoma | | | 5.2% (5) | | | 3.8% (4) | |
| Urological tumor | | | 8.3% (8) | | | 3.8% (4) | |
| Gynecological tumor | | | 6.3% (6) | | | 8.7% (9) | |
| Other solid tumors | | | 2.1% (2) | | | 2.9% (3) | |
| Gastrointestinal tumor | | | 2.1% (1) | | | 1.0% (1) | |
| Head and neck tumor | | | 1.0% (1) | | | 1.0% (1) | |
| Skin tumor | | | 1.0% (1) | | | 1.0% (1) | |
| Recurrence or new disease | | | 18.8% (18/96) | | | 13.2% (12/91) | |
| Months since therapy, median (IQR) | | | 57.5 (14.0; 115.5) | | | 80.0 (23.5; 149.5) | |

*Abbreviations: IG = Intervention Group, CG = Control Group, n = Sample Size, IQR = Interquartile range, m =Meter, kg = Kilogram, BMI = Body Mass Index, WHR = Waist-Hip-Ratio, y = Year, No. = Number*

**Table A5.** “High need” defining criteria for the CFC-nutrition module.

|  | **IG** | | | **CG** | | |
| --- | --- | --- | --- | --- | --- | --- |
| **Criteria** |  | | | | | |
| Poor Dietary Quality - ≤ 40 HEI-EPIC-Score (PRO) | 20.3% (n=26/128) | | | 28.5% (n=37/130) | | |
| BMI < 18.5 | 7.5% (n=10/133) | | | 11.4% (n=15/132) | | |
| Gastrointestinal symptoms (PRO) |  | 28.4%a (n=38/134)^a^ |  |  | 30.1%a (n=40/133)^a^ |  |
| Metabolic syndrome: BMI |  | 20.3% (n=27/133) |  |  | 15.7% (n=21/133) |  |
| Metabolic syndrome: WHR |  | 21.1% (n=24/114) |  |  | 22.8% (n=26/114) |  |
| Metabolic syndrome: Labs |  | 47.0% (n=63/134) |  |  | 42.9% (n=57/133) |  |

^a^ Only available values.

*Abbreviations: IG = Intervention Group, CG = Control Group, n = Sample Size, BMI = Body Mass Index, WHR = Waist-Hip-Ratio, y = Year, No. = Number*

**Table A6.** Intra-group comparison of the outcomes.

| Item | Group | T (n=) | T | Contrast estimator (95% CI) | *p*-value |
| --- | --- | --- | --- | --- | --- |
| Dietary Quality - *HEI-EPIC [Score]* | IG | 1 (127)  2 (93)  3 (87) | 1-2  1-3  2-3 | -4.409 (-6.690 – -2.128)  -2.635 (-4.981 – -0.290)  1.773 (-0.787 – 4.333) | 0.000  0.028  0.174 |
|  | CG | 1 (130)  2 (95)  3 (86) | 1-2  1-3  2-3 | -1.636 (-3.877 – 0.606)  -3.120 (-5.445 – -0.796)  -1.484 (-4.034 – 1.065) | 0.152  0.009  0.253 |
| Mediterranean Dietary Adherence – *MEDAS-FFQ [Score]* | IG | 1 (75)  2 (57)  3 (49) | 1-2  1-3  2-3 | -1.080 (-1.679 – -0.480)  -1.372 (-1.981 – -0.763)  -0.293 (-0.960 – 0.375) | 0.001  0.000  0.381 |
|  | CG | 1 (61)  2 (47)  3 (48) | 1-2  1-3  2-3 | -0.520 (-1.213 – 0.174)  -0.434 (-1.091 – 0.223)  0.086 (-0.670 – 0.841) | 0.140  0.193  0.820 |
| Phase Angle - *Bioelectrical Impedance Analysis [°]* | IG | 1 (94)  2 (79)  3 (59) | 1-2  1-3  2-3 | -0.065 (-0.155 – 0.025)  -0.090 (-0.185 – 0.005)  -0.024 (-0.141 – 0.092) | 0.154  0.064  0.680 |
|  | CG | 1 (88)  2 (63)  3 (62) | 1-2  1-3  2-3 | -0.007 (-0.107 – 0.094)  -0.090 (-0.185 – 0.005)  -0.083 (-0.207 – 0.040) | 0.898  0.064  0.185 |
| Body Mass Index (BMI) *- [kg/m²]* | IG | 1 (131)  2 (70)  3 (87) | 1-2  1-3  2-3 | 0.024 (-0.240 – 0.288)  -0.187 (-0.518 – 0.144)  -0.211 (-0.537 – 0.115) | 0.857  0.266  0.203 |
|  | CG | 1 (131)  2 (58)  3 (88) | 1-2  1-3  2-3 | -0.260 (-0.546 – 0.025)  -0.273 (-0.603 – 0.058)  -0.013 (-0.353 – 0.328) | 0.074  0.105  0.942 |
| EORTC-QLQ-C30 GHS/QoL *[Score]* | IG | 1 (132)  2 (108)  3 (98) | 1-2  1-3  2-3 | -6.566 (-8.660 – -4.473)  -7.833 (-10.425 – -5.242)  -1.267 (-3.848 – 1.314) | 0.000  0.000  0.333 |
|  | CG | 1 (132)  2 (104)  3 (95) | 1-2  1-3  2-3 | -5.156 (-7.289 – -3.023)  -6.703 (-9.336 – -4.070)  -1.547 (-4.170 – 1.077) | 0.000  0.000  0.245 |
| EORTC-QLQ-C30 Fatigue *[Score]* | IG | 1 (132)  2 (110)  3 (99) | 1-2  1-3  2-3 | 9.074 (4.683 – 13.465)  11.145 (6.559 – 15.731)  2.071(-2.342 – 6.485) | 0.000  0.000  0.356 |
|  | CG | 1 (132)  2 (104)  3 (96) | 1-2  1-3  2-3 | 8.628 (4.125 – 13.130)  11.523 (6.862 – 16.184)  2.895 (-1.625 – 7.415) | 0.000  0.000  0.208 |
| Nutrition-related Barriers – total score *[Score]* | IG | 1 (102)  2 (95)  3 (82) | 1-2  1-3  2-3 | 0.099 (-0.067 – 0.265)  0.099 (-0.061 – 0.259)  -0.001 (-0.127 – 0.126) | 0.230  0.221  0.993 |
|  | CG | 1 (95)  2 (78)  3 (76) | 1-2  1-3  2-3 | 0.044 (-0.133 – 0.221)  0.088 (-0.083 – 0.258)  0.043 (-0.094 – 0.181) | 0.615  0.307  0.535 |

*Abbreviations: IG = Intervention Group, CG = Control Group, CI = Confidence interval, T = Time Point of Measurement, BMI = Body Mass Index*

**References**

1.	Hopewell S, Chan A-W, Collins GS, Hróbjartsson A, Moher D, Schulz KF, et al. CONSORT 2025 statement: updated guideline for reporting randomised trials. BMJ. 2025;389:e081123.

2.	Hoffmann TC, Glasziou PP, Boutron I, Milne R, Perera R, Moher D, et al. Better reporting of interventions: template for intervention description and replication (TIDieR) checklist and guide. BMJ : British Medical Journal. 2014;348:g1687.

3.	von Rusten A IA-K, Boeing H, Flothkotter M. Begutachtetes Original-Die Bewertung der Lebensmittelaufnahme mittels eines' Healthy Eating Index'(HEI-EPIC). Ernahrungs-Umschau. 2009;56(8):450.

4.	Ruesten A. Die Bewertung der Lebensmittelaufnahme mittels eines ,Healthy Eating Index‘(HEI-EPIC). Ernährungs Umschau. 2009;8:450–6. 2009.

5.	Guenther PM, Casavale KO, Reedy J, Kirkpatrick SI, Hiza HA, Kuczynski KJ, et al. Update of the Healthy Eating Index: HEI-2010. J Acad Nutr Diet. 2013;113(4):569-80.

6.	V. Ruesten A. Die Bewertung der Lebensmittelaufnahme mittels eines ,Healthy Eating Index‘ (HEI-EPIC). Ernährungs Umschau. 2009;8:450-6.

7.	Quidde J, et al. Improved Nutrition in Adolescents and Young Adults after childhood cancer: INAYA—A pilot study. Cancer Survivorship Symposium: Advancing Care and Research; January: J Clin Oncol; 2016.

8.	Kluthe B. Freiburger Ernaehrungsprotokoll. 2023 [Available from: <www.ernaehrung.de/static/pdf/freiburger-ernaehrungsprotokoll.pdf>

9.	(DGE) GNS. 10 Guidelines of the German Nutrition Society (DGE) for a Wholesome Diet 2024 [cited 2024 24/07/2024]. Available from: <https://www.dge.de//fileadmin/dok/english/dge-recommendations/information-sheet-DGE-recommendations-en.pdf>.

10.	Barbosa-Silva MC, Barros AJ. Bioelectrical impedance analysis in clinical practice: a new perspective on its use beyond body composition equations. Curr Opin Clin Nutr Metab Care. 2005;8(3):311-7.

11.	Norman K, Stobäus N, Pirlich M, Bosy-Westphal A. Bioelectrical phase angle and impedance vector analysis--clinical relevance and applicability of impedance parameters. Clin Nutr. 2012;31(6):854-61.

12.	Norman K, Stobäus N, Zocher D, Bosy-Westphal A, Szramek A, Scheufele R, et al. Cutoff percentiles of bioelectrical phase angle predict functionality, quality of life, and mortality in patients with cancer. Am J Clin Nutr. 2010;92(3):612-9.

13.	de Luis DA, Aller R, Romero E, Dueñas A, Perez Castrillon JL. Relation of phase angle tertiles with blood adipocytokines levels, insulin resistance and cardiovascular risk factors in obese women patients. Eur Rev Med Pharmacol Sci. 2010;14(6):521-6.

14.	Gonzalez MC, Barbosa-Silva TG, Bielemann RM, Gallagher D, Heymsfield SB. Phase angle and its determinants in healthy subjects: influence of body composition. Am J Clin Nutr. 2016;103(3):712-6.

15.	Kyle UG, Bosaeus I, De Lorenzo AD, Deurenberg P, Elia M, Manuel Gómez J, et al. Bioelectrical impedance analysis-part II: utilization in clinical practice. Clin Nutr. 2004;23(6):1430-53.

16.	Hebestreit K, Yahiaoui-Doktor M, Engel C, Vetter W, Siniatchkin M, Erickson N, et al. Validation of the German version of the Mediterranean Diet Adherence Screener (MEDAS) questionnaire. BMC Cancer. 2017;17(1):341.

17.	Estruch R, Ros E, Salas-Salvadó J, Covas MI, Corella D, Arós F, et al. Retraction and Republication: Primary Prevention of Cardiovascular Disease with a Mediterranean Diet. N Engl J Med 2013;368:1279-90. N Engl J Med. 2018;378(25):2441-2.

18.	International WCRF. Diet, nutrition, physical activity and cancer: a global perspective: a summary of the third expert report: World Cancer Research Fund International; 2018.

19.	Keller. Zur Validität des Transtheoretischen Modells: Eine Untersuchung zur Veränderung des Ernährungsverhaltens. Dissertation. Universität Marburg 1998.

20.	Prochaska JO, DiClemente CC. Transtheoretical therapy: Toward a more integrative model of change. Psychotherapy: Theory, Research & Practice. 1982;19(3):276-88.

21.	Warschburger P. Neuere Modelle zur Veränderung. In: Warschburger P, editor. Beratungspsychologie. Berlin, Heidelberg: Springer Berlin Heidelberg; 2009. p. 81-103.

22.	Obesity: preventing and managing the global epidemic. Report of a WHO consultation. World Health Organ Tech Rep Ser. 2000;894:i-xii, 1-253.

23.	Aaronson NK, Ahmedzai S, Bergman B, Bullinger M, Cull A, Duez NJ, et al. The European Organization for Research and Treatment of Cancer QLQ-C30: a quality-of-life instrument for use in international clinical trials in oncology. J Natl Cancer Inst. 1993;85(5):365-76.

24.	Fayers PM, Aaronson N, Bjordal K. EORTC QLQ-C30 scoring manual2001.

25.	Bower JE. Cancer-related fatigue--mechanisms, risk factors, and treatments. Nat Rev Clin Oncol. 2014;11(10):597-609.

26.	Matias M, Baciarello G, Neji M, Di Meglio A, Michiels S, Partridge AH, et al. Fatigue and physical activity in cancer survivors: A cross-sectional population-based study. Cancer Med. 2019;8(5):2535-44.

27.	Storey DJ, Waters RA, Hibberd CJ, Rush RW, Cargill AT, Wall LR, et al. Clinically relevant fatigue in cancer outpatients: the Edinburgh Cancer Centre symptom study. Annals of Oncology. 2007;18(11):1861-9.

28.	Landis BN, Welge-Luessen A, Brämerson A, Bende M, Mueller CA, Nordin S, et al. "Taste Strips" - a rapid, lateralized, gustatory bedside identification test based on impregnated filter papers. J Neurol. 2009;256(2):242-8.

29.	Czesnik D, Just T, Damm M, Landis BN, Gudziol H, Manzini I, et al. Riech- und Schmeckstörungen. Stuttgart: Georg Thieme Verlag KG; 2009. Available from: <http://www.thieme-connect.de/products/ebooks/book/10.1055/b-002-33686>.
