# Supplementary material for: Dietary intervention among young cancer survivors within the CARE for CAYA program
Source: J Cancer Surviv. 2026 Mar 26;20(Suppl 2):1754–66. doi: 10.1007/s11764-026-01997-4 (PMC13744525; doi:10.1007/s11764-026-01997-4)
Supplement: Supplementary file 2 [file 11764_2026_1997_MOESM2_ESM.docx]

**Appendix B: Supplementary materials**: Figures

**outlier*

**Figure A1.** Changes in different food groups (HEI-EPIC).

**Figure A2.** Barriers and facilitators.

The median of the ten barriers and facilitators decreased in IG and CG at T3 (Table 4). To the question "If I eat a healthy diet, i.e. a varied diet with a high proportion of plant foods...", the three most commonly reported (extremely and very important) facilitators that increased to T3 (both groups) were on the mental basis: "I simply feel better mentally", "I will be happier and more balanced" and "I have more energy". The three most commonly reported barriers that decreased to T3 (both groups) were on the physical basis: "I feel very restricted when eating", "I don`t get really full" and "I have to eat things I don’t like". Facilitators and barriers decreased slightly more in the IG, as reflected in the median.

**Figure A3.** TTM-Stages.

The TTM-stages were also assessed. For the question "Do you eat a healthy diet most of the time, i.e. a varied diet with a high proportion of plant foods?", the most common stage in IG and CG at T1 and T3 was at the highest stage 5 (Maintenance). In the IG, the proportions (with 54.8% and 67.9%) were higher at T1 and T3 than in the CG (with 37.6 and 54.7). Both groups could improve their maintains with the respect to T1 to T3 (Figure 3). No significant improvements between the group could be found at T3 (*p* = 0.454). In more detail, the improvements from T1 to T3 (*p* = 0.033) and from T2 to T3 (*p* = 0.013) in CG and from T1 to T3 (*p* = 0.009) in the IG were statistically significant.

**Figure A4.** Satisfaction with the CFC-nutrition module (T3).
